# Regulation and Maintenance of Vascular Tone and Patency in Cardiovascular Health and Disease

**DOI:** 10.1155/2012/396369

**Published:** 2012-04-09

**Authors:** John W. Calvert, Christopher G. Kevil, Matthew R. Spite

**Affiliations:** ^1^Division of Cardiothoracic Surgery, Department of Surgery, Carlyle Fraser Heart Center, Emory University School of Medicine, 550 Peachtree Street NE, Atlanta, GA 30308, USA; ^2^Departments of Pathology, Molecular and Cellular Physiology, and Anatomy and Cell Biology, LSU Health Sciences Center Shreveport, Shreveport, LA 71103, USA; ^3^Division of Cardiovascular Medicine, University of Louisville, Louisville, KY 40202, USA

Despite numerous advances in health care practices, cardiovascular disease still remains the leading cause of morbidity and mortality worldwide. Perhaps the most important consequence of cardiovascular disease is the interruption of blood flow to organs such as the heart and brain, resulting in the clinical presentation of a heart attack or stroke. As such, the regulation of vascular tone and the maintenance of vascular patency are vital for the preservation of cardiovascular health. Central to this process is the vascular endothelium. The endothelium is vital for the regulation of vascular tone and the maintenance of vascular homeostasis, as it releases factors such as nitric oxide, hydrogen sulfide, endothelial-dependent hyperpolarizing factor, and prostacyclin that induce vasodilatation and keep the vasculature free of obstructions [1]. Therefore, it should be of no surprise that a number of the risk factors for the development of cardiovascular disease, such as hypertension, hypercholesterolemia, diabetes, smoking, ageing, and atherosclerosis, correlate with an impaired endothelium [2]. This impairment, termed endothelial dysfunction, is characterized by the reduction in the bioavailability of vasodilators, particularly nitric oxide, and/or an increase in endothelium-derived contracting factors [3]. The resulting imbalance leads to an impairment of endothelium-dependent vasodilation, as well as a compromised state of endothelial activation characterized by proinflammatory, proliferative, and procoagulatory conditions that favor all stages of atherogenesis [4]. Therefore, it is critically important to develop and implement therapeutic strategies that will combat endothelial dysfunction in an effort to reduce the mortality and morbidity associated with cardiovascular diseases. However, before therapeutic strategies can be implemented it is important to have a clear understanding of the pathological mechanisms that lead to the endothelial dysfunction in the first place.

This special issue of highlights the latest findings related to the role of endothelial dysfunction in cardiovascular disease and comprises five review articles and one original report. The first three articles share the common theme of endothelial dysfunction in diabetes. Each paper offers unique insights into the mechanisms underlying diabetes-induced changes in endothelial biology and highlights specific pathways that could be novel therapeutic targets.

The review article in this series by S. D. Funk et al. discusses the mechanisms that contribute to the development of atherosclerosis in diabetics. This paper highlights the central role of hyperglycemia in promoting endothelial dysfunction and discusses how cellular and animal studies have translated to clinical development of therapeutics targeting endothelial dysfunction in diabetes. The next paper by A. Sharma et al. highlights the importance of alterations in endothelium-derived nitric oxide (EDNO) production in the pathogenesis of vascular complications in both type 1 and type 2 diabetes. The interrelationship between reactive oxygen species (ROS) and EDNO is described, with emphasis on the cellular and molecular mechanisms that give rise to altered NO bioavailability in diabetes. In their paper, G. K. Kolluru et al. emphasize the importance of angiogenesis in diabetes and chronicle studies that have yielded mechanistic insights into the development of endothelial dysfunction in diabetes. This comprehensive paper, entitled “*Endothelial dysfunction and diabetes: effects on angiogenesis, vascular remodeling, and wound healing*,” discusses how ROS and deficiencies in NO bioavailability contribute to altered angiogenesis in diabetes. Therapeutic management of angiogenesis in diabetes is also discussed.

In another review article in this series, M. Murakami provides a comprehensive overview of the mechanisms contributing to the active maintenance of the vasculature. The role of growth factors and cytokines in mediating endothelial and mural cell maintenance is described, with an analysis of how genetic alterations in these pathways give rise to blood vessel abnormalities and susceptibility to vascular disease. The molecular and cellular mechanisms leading to restenosis and the potential for new therapeutics targeting vascular smooth muscle cell (VSMC) proliferation are the topic of a review article in this series. L. Denes et al. highlight the importance of VSMC phenotype switching and proliferation in vascular disease and discuss the clinical diagnosis and management of restenosis.

The original report by I. Liesmaa et al. is entitled “*Bradykinin type-2 receptor expression correlates with age and is subjected to transcriptional regulation*.” This study demonstrates that bradykinin type-2 receptor (BK-2R) mRNA is positively correlated with age, but is significantly reduced in human patients with idiopathic dilated cardiomyopathy (IDC) compared with healthy individuals. The study further examines the relationship between polymorphisms in the BK-2R promoter and the presence of IDC and coronary heart disease.
